# High-Resolution Differentiation of Enteric Bacteria in Premature Infant Fecal Microbiomes Using a Novel rRNA Amplicon

**DOI:** 10.1128/mBio.03656-20

**Published:** 2021-02-16

**Authors:** J. Graf, N. Ledala, M. J. Caimano, E. Jackson, D. Gratalo, D. Fasulo, M. D. Driscoll, S. Coleman, A. P. Matson

**Affiliations:** a University of Connecticut, Department of Molecular and Cell Biology, Storrs, Connecticut, USA; b UConn Health, Department of Pediatrics, Farmington, Connecticut, USA; c UConn Health, Department of Medicine, Farmington, Connecticut, USA; d Shoreline Biome, Farmington, Connecticut, USA; e Pattern Genomics, Madison, Connecticut, USA; f Connecticut Children’s Medical Center, Division of Neonatology, Hartford, Connecticut, USA; University of Hawaii at Manoa

**Keywords:** 16S rRNA, long-read sequencing, microbial community, microbiome, human infant, neonates, bacterial strains

## Abstract

Identifying and tracking microbial strains as microbiomes evolve are major challenges in the field of microbiome research. We utilized a new sequencing kit that combines DNA extraction with PCR amplification of a large region of the rRNA operon and downstream bioinformatic data analysis. Longitudinal microbiome samples of coadmitted twins from two different neonatal intensive care units (NICUs) were analyzed using an ∼2,500-base amplicon that spans the 16S and 23S rRNA genes and mapped to a new, custom 16S-23S rRNA database. Amplicon sequence variants (ASVs) inferred using DADA2 provided sufficient resolution for the differentiation of rRNA variants from closely related but not previously sequenced *Klebsiella*, Escherichia coli, and *Enterobacter* strains, among the first bacteria colonizing the gut of these infants after admission to the NICU. Distinct ASV groups (fingerprints) were monitored between coadmitted twins over time, demonstrating the potential to track the source and spread of both commensals and pathogens. The high-resolution taxonomy obtained from long amplicon sequencing enables the tracking of strains temporally and spatially as microbiomes are established in infants in the hospital environment.

## INTRODUCTION

The gut microbiome helps establish and maintain critical systems for lifelong health, including the establishment and maintenance of the gut mucosal barrier, the endocrine system, nutritional metabolism, and pathogen defense (1). An understanding of factors that influence the establishment of the gut microbiome requires a detailed understanding of the diverse array of microbial strains involved during development (2). Most publications to date rely on sequencing a short region of the 16S rRNA gene that typically allows identification (ID) to the family or genus level but only in rare cases to the species or strain level (3). The importance of strain-level identification is exemplified by Escherichia coli, which includes benign strains as well as pathogenic strains, such as serotypes O104:H4 and O113:H21, which carry the Shiga toxin gene responsible for hemorrhagic diarrhea (4). Since strains belonging to the same species can range from benign to pathogenic, strain-level identification is an important barrier that needs to be overcome in order to decipher links between the microbiome and human health.

**FIG 1:**
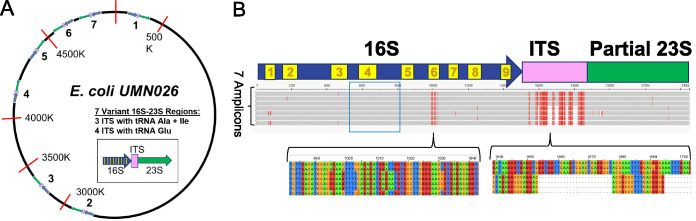
Multiple StrainID amplicons generated from an E. coli genome. (A) Circle representing the E. coli UMN026 genome, with the seven 16S-23S rRNA operons shown. The internally transcribed spacer (ITS) between the genes is represented as a pink box, the 16S rRNA gene is a blue arrow, and the 23S rRNA gene is represented by a green box. (B) Multiple-sequence alignment of the 7 amplicon regions from E. coli UMN026 (GenBank accession number NC_011751). The blue arrow with the numbered yellow boxes represents the 16S rRNA gene in the aligned sequences, with the numbered yellow boxes indicating the locations of the variable regions. The red hash marks indicate variant bases. The white gaps indicate deletions. Three of the seven 16S-23S amplicons contain two tRNA genes, tRNA Ala and tRNA Ile, in the ITS, while the four shorter ITSs contain a single tRNA Glu. The base-level alignments at the bottom detail some of the key variant sites in the V6 region and in the ITS region indicated by brackets. The blue box surrounding 16S V4 highlights an invariant sequence across all 7 amplicons that includes bases 513 to 806 in the 16S V4 region that is a common 16S target amplicon region.

DNA sequencing techniques are useful for both high- and low-resolution taxonomic identification of bacteria in microbiome samples. The ability to differentiate closely related bacteria using next-generation sequencing methods depends on the read length and quality of the DNA sequence obtained as well as the taxonomic curation and depth of content of the reference database used for mapping. In addition, the accuracy of the software algorithms used to compare the raw sequence data with reference databases or infer amplicon sequence variants (ASVs) may vary (5). In general, increasing the amplicon read length is useful in obtaining higher-resolution taxonomy for bacteria in a microbiome (6). Since the genome of a single bacterium contains multiple 16S-23S rRNA genes that can vary in sequence, the combination and relative abundance of 16S-23S variants can be useful in determining bacterial taxonomy at the species level and lower. For example, the E. coli UMN026 genome contains seven copies of the 16S and 23S genes, separated by a short internally transcribed spacer (ITS) sequence that can vary in length (Fig. 1). The seven copies produce seven distinct amplicons for a single genome, which vary in length and sequence. Generating multiple amplicons per genome therefore presents the opportunity to use the combination as a “fingerprint” profile to identify a given strain, even if closely related strains share one or more 16S-23S variants. In this study, a high-resolution ∼2,500-bp 16S-ITS-23S amplicon (StrainID) that utilized highly conserved annealing sites (7–9) was used to explore whether 16S-23S amplicons, and combinations thereof, from *Klebsiella* spp., E. coli, and *Enterobacter* spp. present in premature infant fecal samples could be used to identify and track individual strains during microbiome development. ASVs were inferred using DADA2 (10) and used to confirm the presence of previously unsequenced strains and determine whether these novel organisms were present in different longitudinal samples and individuals. The results demonstrate that 16S-23S StrainID amplicon fecal microbiome sequencing generates multiple distinct ASVs for individual bacterial strains and that the combination and relative abundance of ASVs can be used as a fingerprint to track previously unsequenced species longitudinally and across infants in the neonatal intensive care unit (NICU).

## RESULTS

### The StrainID amplicon enables species- and strain-level detection of pathogens in fecal microbiomes from premature infants.

**FIG 2:**
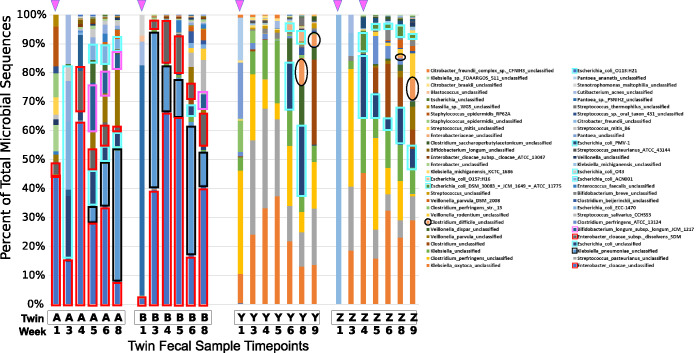
Twin pair A/B and Y/Z bacterial community profiles. Fecal samples from two pairs of twins were profiled from week 1 to week 8 (twin pair A/B) or week 9 (twin pair Y/Z) after birth. Twins A and B were admitted to a NICU in a different hospital than twins Y and Z. Samples were obtained during the week indicated on the *x* axis. Results are shown for each sample as a “100% stacked column,” with the *y* axis box size indicating the percentage of the total sequences. Pink triangles at the top indicate antibiotic treatment prior to sample collection. The following specific taxa are highlighted with the indicated colored borders: Enterobacter cloacae, Klebsiella pneumoniae, E. coli taxa, Bifidobacterium longum, and Clostridium difficile.

Sequential fecal samples from two sets of premature twins, born at gestational ages of 29 and 30 weeks, the first set delivered by vaginal delivery and the second set by cesarean section (C-section), with each set being cared for in a different NICU, were collected, and 16S-ITS-23S amplicons were analyzed using SBanalyzer. Figure 2 demonstrates that infant twins A and B shared *Klebsiella* spp., E. coli, and *Enterobacter* spp., which comprised more than half of the reads recovered from every sample and allowed us to monitor the temporal colonization dynamics. For example, Enterobacter cloacae strains, a common pathogen in NICU environments (11), were present at all time points, while Klebsiella pneumoniae strains appeared in twin A at week 4 and twin B at week 3 and remained a significant component of the overall read count subsequently in both twins. Microbial diversity increased over time, with the appearance of the common digestive tract residents *Veillonella* spp. and *Bifidobacterium* spp. at later time points. The important gut symbiont Bifidobacterium longum (12) was established early in twin A, present in a small quantity at week 3, remaining at over 5% of reads through week 8. Bifidobacterium longum was established in twin B only at the final week 8 time point. For the first 4 to 5 weeks, both twin pairs received a diet consisting primarily of human milk, an important source of oligosaccharides that benefit the growth of *Bifidobacterium* spp. (13).

As with the twin A/B pair, there was a high degree of similarity within the bacterial species present in the twin Y/Z pair, but there were striking differences between the sets of twins. The week 1 sample for twin Z was excluded from further analysis due to a low read count. For all other samples in twins Y and Z, Klebsiella oxytoca and Clostridium perfringens were present at high levels, both of which have been associated with necrotizing enterocolitis (NEC) in preterm infants (14, 15). Streptococcus pasteurianus, a group D *Streptococcus* sp. reported to be a cause of sepsis and meningitis in twin infants (16), was absent following empirical treatment with ampicillin and gentamicin for both twins Y and Z; however, it reappeared in twin Z by week 5 and was present at all subsequent time points. It is possible that the decreased biomass and associated lower read count interfered with the detection of S. pasteurianus and other bacteria. A similar pattern was observed for Clostridium perfringens. E. coli appeared in twin Z by week 4 concurrent with antibiotic treatment but was not present in twin Y until week 6, an indication that antibiotics may have facilitated more rapid colonization by reducing microbial competition.

As premature infant samples harbor a low-complexity microbiome, relatively few reads were needed for an accurate representation of the most abundant organisms. Rarefaction plots for the microbiome samples illustrate that for most samples, a few hundred reads were sufficient to account for most of the ASVs present in any given sample, even though each species was represented by multiple ASVs (see Fig. S1 in the supplemental material). The samples from weeks 1 through 3 and time points following antibiotic treatments yielded relatively few reads, which is likely due to either the samples largely being meconium stool that develops during the fetal period or antibiotics depleting populations of microbes, resulting in low bacterial biomass. It is noteworthy that pathogens such as C. difficile and K. pneumoniae found commonly within the hospital environment were among the first bacteria to colonize the preterm infant gut, and these were seen at later time points.

10.1128/mBio.03656-20.1 FIG S1 Rarefaction plot for microbiome samples. Rarefactions plots were generated for each microbiome sample using QIIME2 (42). Ten iterations were calculated for up to 1,000 reads per sample. Box-whisker plots for each sampling depth were plotted. Download FIG S1, EPS file, 0.3 MB. Copyright © 2021 Graf et al. 2021 Graf et al. https://creativecommons.org/licenses/by/4.0/ This content is distributed under the terms of the Creative Commons Attribution 4.0 International license.

10.1128/mBio.03656-20.2 FIG S2A Taxonomic tree of *Klebsiella* 16S and 16S-23S StrainID amplicon sequences. Amplicon sequences from Athena database were extracted using the 27f StrainID forward and either the 1492r (16S V1V9) or StrainID Reverse (V1V9-ITS-23S) sequences from Materials and Methods. The sequences were aligned in Geneious Prime version 2020.1 using Clustal Omega (30) and the phylogeny inferred using RAxML (31). The tree was annotated in iTOL (32). Download FIG S2A, EPS file, 1.2 MB. Copyright © 2021 Graf et al. 2021 Graf et al. https://creativecommons.org/licenses/by/4.0/ This content is distributed under the terms of the Creative Commons Attribution 4.0 International license.

10.1128/mBio.03656-20.3 FIG S2B StrainID phylogeny. Download FIG S2B, EPS file, 1.2 MB. Copyright © 2021 Graf et al. 2021 Graf et al. https://creativecommons.org/licenses/by/4.0/ This content is distributed under the terms of the Creative Commons Attribution 4.0 International license.

These initial mapping data demonstrate that high-resolution taxonomic assignments enable the tracking of specific gut bacteria, including important pathogens, during the colonization of the premature infant gut within the NICU environment at species- and even strain-level resolution. As bacterial communities are established and diversify within individuals, the ability to track the ebb and flow of specific strains provides an important tool for correlating bacterial colonization with health status. The mapping data also revealed that there were high numbers of reads that were not classified to lower than the genus level.

### DADA2-inferred ASV sequence resolution enables differentiation of closely related *Klebsiella* spp.

Sequencing-related methods to track E. cloacae infections have been shown to be useful in tracking strain transmission (17), but given the number of “unclassified” sequences in our samples and the possibility that strains can differ by only a few bases, it was clear that comparing closely related *Enterobacter* species, E. coli, and *Klebsiella* species strains by mapping amplicon reads to the 16S-23S (Athena) database alone would be insufficient for strain tracking. To move beyond the limitations of mapping individual reads to reference sequences, we used DADA2 to error correct and infer ASVs from the StrainID sequencing data for these genera (10). The ASV sequences can be compared not only to the reference genomes but also against each other to determine and ascertain if the sample contains novel ASVs that are different from the novel ASVs in other samples. Since almost every sample for both sets of twins contained *Klebsiella*, it was selected as an initial test case for novel strain differentiation.

**FIG 3:**
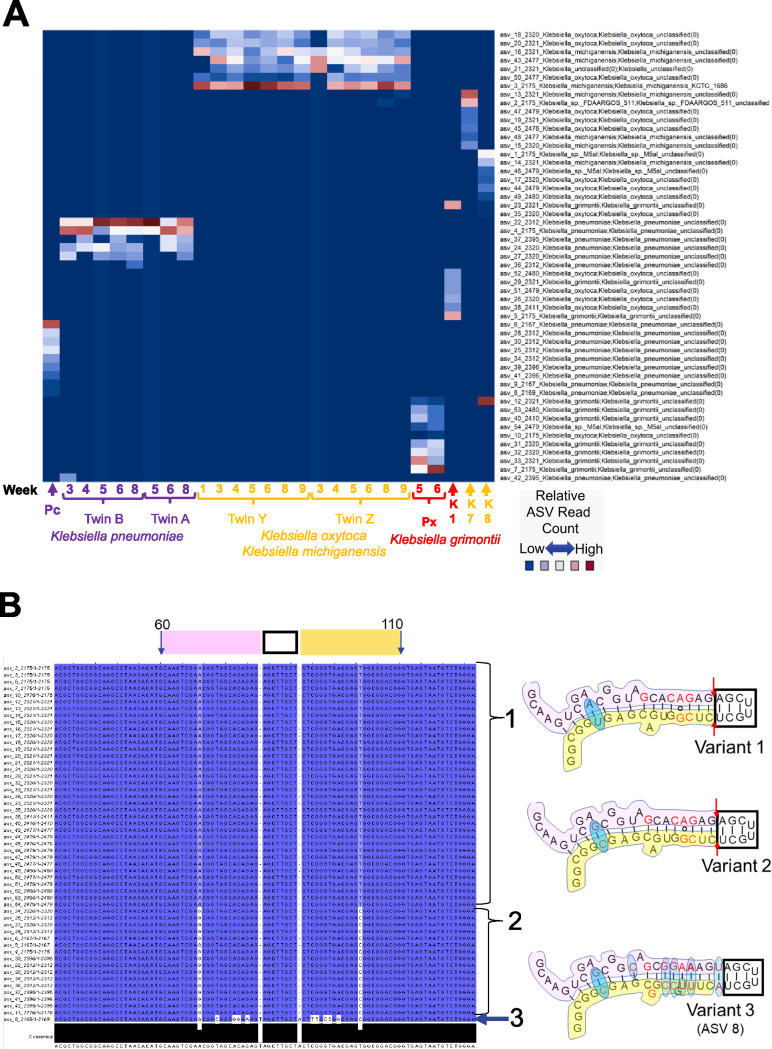
*Klebsiella* amplicon sequence variant fingerprints. (A) Relative-abundance heat map of ∼2,100- to 2,500-base StrainID amplicon sequence variants (ASVs). Samples sharing *Klebsiella* strains have similar ASV fingerprint patterns. On the *x* axis, sample IDs are indicated by brackets, and numbers indicate the week since birth when the sample was obtained. Individual samples are indicated by arrows. “Pc” and “Px” indicate fecal sample ASVs from unrelated preterm infants, classified as K. pneumoniae and *K. grimontii*, respectively. K1, K7, and K8 represent ASVs from *Klebsiella* isolates from unrelated preterm infants who developed necrotizing enterocolitis. Reads classified by SBanalyzer as *Klebsiella* were analyzed using DADA2, resulting in the 54 *Klebsiella* ASVs listed on the *y* axis. The ASV number is followed by the four-digit ASV length and the taxonomy assigned for that ASV by SBanalyzer. Each coordinate representing an ASV in a sample was colored according to the relative number of reads. Possible species-level taxonomic assignments for the fingerprint in each sample based on the best mapping results on the *y* axis are indicated by the label colors on the *x* axis for K. pneumoniae, K. oxytoca/*K. michiganensis*, and *K. grimontii*. (B) *Klebsiella* ASVs in V1 maintain a conserved stem-loop structure. All 54 *Klebsiella* ASVs were aligned in Jalview (40), with each base numbered according to the alignment and shaded by percent identity. Dark blue variant bases match the most abundant variations, and white indicates a rare variant base. Bases 60 to 110 are indicated at the top of the alignment, part of the 16S V1 region containing a conserved stem-loop based on the E. coli 2-D structure (41). The 5′ stem, loop, and 3′ stem are marked above the alignment by pink, black, and yellow boxes, respectively. Only three variant structures were identified at the V1 loop, shown at the right. Brackets 1 and 2 indicate the ASVs with variants 1 and 2, respectively. Arrow 3 indicates ASV 8, the only ASV with a variant 3 sequence. The pink, black, and yellow regions of the variant structures match the sequence in the alignment window. The red bases in the structures indicate *Klebsiella*-specific sequences common to these variants that differ from the E. coli structure. The red arrows in variants 1 and 2 indicate a deletion from the E. coli and variant 3 structures. The blue ovals indicate base variations between the aligned *Klebsiella* ASVs.

The DADA2 analysis of all of the reads identified as *Klebsiella* resulted in 54 ASVs. Subsets of ASVs representing individual strain “fingerprints” were present in each of the 24 infant fecal samples and 3 *Klebsiella* isolates, K1, K7, and K8 (Fig. 3A). *Klebsiella* genomes typically contain 8 16S-23S operons, and the analysis of the amplicons from 3 pure cultures revealed the presence of a unique ASV fingerprint comprised of 7 different ASVs for each strain. The differences in relative abundance suggest that one ASV may be present as multiple copies.

Analysis of the *Klebsiella* ASV fingerprint patterns revealed the long-term colonization of premature infants by the same strain and overall similarities between strains isolated between the twin pairs. Twins A and B shared one *Klebsiella* ASV pattern, and twins Y and Z shared another pattern, suggesting that each pair was colonized by one strain and that these strains colonized each twin for at least 3 to 7 weeks. The ASVs in twins A and B and infant “Pc” were taxonomically classified as Klebsiella pneumoniae, although the ASVs were not 100% identical to strains in the Athena database. The ASV fingerprint in twins A and B was distinct from the ASV fingerprint in sample Pc, indicating that twins A and B and sample Pc harbored distinct K. pneumoniae strains.

Twins Y and Z shared an ASV fingerprint over many weeks that mapped most closely to either K. oxytoca or the closely related species Klebsiella michiganensis. The twin Y/Z ASVs were almost completely different from the ASVs for other *Klebsiella* samples, including samples from infant “Px” and isolates K1, K7, and K8. *Klebsiella* ASVs from fecal samples obtained from infant Px 5 and 6 weeks after birth contained ASVs that were classified as an unknown Klebsiella grimontii strain by mapping to the Athena database. K. grimontii is a species related to but distinct from K. oxytoca and K. michiganensis (18). The isolates K1, K7, and K8 were cultured from fecal samples obtained from nontwin preterm infants with NEC (14), and each contained a unique ASV pattern.

The ASV fingerprints convincingly demonstrate that StrainID ASVs can be used to differentiate closely related *Klebsiella* spp. and may have utility in monitoring pathogenic strains in conjunction with other determinants of virulence in multiple individuals over time.

### ASVs reflect constraints of 2-dimensional rRNA structure, an independent confirmation of DADA2 ASV accuracy at the single-base level.

The ASVs produced from the StrainID sequence data were investigated further to explore the degree to which the sequence variations were underpinned by the biology of the 16S rRNA molecule. As an example, we depict the variation across all 54 *Klebsiella* ASVs in a portion of the 16S gene V1 region in Fig. 3B. The two-dimensional (2-D) structures show that the variations in the V1 stem-loop between bases 60 and 110 were not random and reveal that the sequence variants fit with the hypothesis that they are evolutionarily constrained to maintain 16S function (19). This result is consistent with the observed variation in the ASVs, evidence that the DADA2-derived ASVs are an accurate representation of *Klebsiella* genomic variation and not the result of PCR or sequencing artifacts. Although the ASVs originated in different individuals and time points, the overall variation at the V1 stem-loop was limited to three areas. The tip of the V1 loop, which was invariant in all 54 ASVs, is part of 16S helix 6, which is exposed to solution in the crystal structure and may be important in the regulation of ribosome function (20). Variants 1 and 2 contained a double deletion, adjacent to the tip, which eliminated a base pair compared to E. coli but maintained an equal-length sequence on both sides of the stem. Variant 3 had a total of 13 bases that differed from variant 1 and were represented by a single ASV (ASV 8), of which 12 base changes maintained base pairing and the 13th was an A/G substitution at a bulge that conserved a purine at that site. For each variant 1 region inferred for the 54 *Klebsiella* ASVs, overlaying the base-level variation observed in this 100-base window revealed that the ASVs generated by DADA2 were consistent with maintaining the 16S rRNA structure. The observation that the base-level variation in the ASVs is consistent with the maintenance of the 2-dimensional structure of the 16S rRNA molecule is strong evidence that the ASVs generated by DADA2 are representative of the true genomic variation of the bacterial genes across all ∼2,500 bases of the StrainID amplicon.

Investigation of *Klebsiella* species demonstrated that long amplicons can be used to differentiate novel, closely related species and strains in a sample set. Similar methods were used to determine the relatedness of *Enterobacter* and E. coli in an expanded infant fecal microbiome sample set.

### *Enterobacter* ASV fingerprints enable ID and tracking of strains across samples.

**FIG 4:**
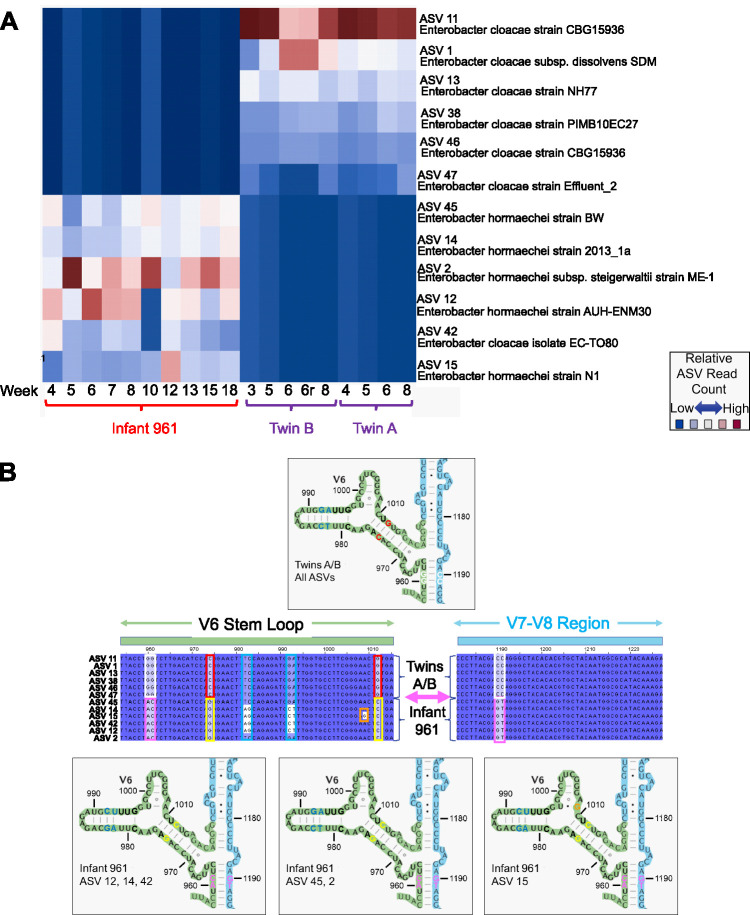
*Enterobacter* amplicon sequence variant fingerprints. (A) *Enterobacter* ASVs were inferred from longitudinal samples at the indicated weeks from reads classified as *Enterobacter* by SBanalyzer from twins A and B and an additional individual, infant 961, indicated by brackets. All ASVs were mapped to the NCBI database, and the closest-matching taxonomies were selected and are displayed on the *y* axis to the right. The relative number of each of the ASVs in each sample is indicated by shading. Points 6 and 6r in twin B are technical repeats of the week 6 fecal sample. (B) *Enterobacter* ASV mapping to the 2-D 16S rRNA structure. The 12 *Enterobacter* ASVs inferred from twin A, twin B, and infant 961 using DADA2 were aligned against each other in Jalview (40), with each base numbered according to the alignment and shaded by percent identity. Dark blue variant bases match the most abundant variations, and white indicates a rare variant base. The stem-loop rRNA structures are based on E. coli (41). The selected sequences shown in the middle were mapped to the 16S gene structure from bases 955 to 1015 (the V6 stem-loop [green box above the sequence]) and bases 1180 to 1230 (region between V7 and V8 [light blue box above the sequence]). Identical sequences across all ASVs are shown in black text with dark blue highlighting. Variant sequences are shaded and boxed with different colors, indicating the text color of the variant bases in the corresponding structures. The structure above the alignments represents all twin A/B ASV structures, which are identical in the regions shown. There are three variant structures in this sequence shared within the infant 961 samples, shown below the alignments. The variations in the V7-V8 region from bases 1190 to 1191 form a base-paired structure with the V6 region ∼200 bases away at bases 960 to 961.

In Fig. 4A, *Enterobacter* ASVs were inferred from twin A, twin B, and infant 961 samples, all of which contained significant quantities of *Enterobacter* at multiple time points. The resulting ASVs were mapped to the NCBI database using BLAST, which resulted in six ≥99%, but nonidentical, matches to a variety of strains for each sample. While *Enterobacter* strains typically contain 8 rRNA operons, a scan of *Enterobacter* strains revealed that one or more regions may be an exact duplicate within a genome, resulting in 6 to 8 amplicons per genome. For each individual, the same sets of ASVs persisted over the weeks sampled, evidence that the novel variants are not artifacts. The reproducibility of ASVs within a sample is shown by comparison of twin B technical replicates 6 and 6r, which yielded identical ASVs in similar relative proportions. Twins A and B contained the same overlapping set of 6 ASVs, starting 1 week apart and continuing through week 8. These twins, admitted to the same NICU at the same time, were apparently colonized a week apart by the same Enterobacter cloacae strain. In contrast to the twins, infant 961 contained ASVs that mapped primarily to a variety of regions from Enterobacter hormaechei strains. Antibiotic treatment at week 17 resulted in the loss of all *Enterobacter* ASVs, so that time point is not represented. However, the same ASVs reappeared by week 18, indicating that either *Enterobacter* was reduced to undetectable levels but not completely eliminated from the gut or reinfection of the same strain occurred. In Fig. 4B, *Enterobacter* variants inferred from the different samples were aligned against each other and mapped back to the 2-D 16S rRNA structure. Because the base changes were complementary and maintained across many different time points, it is likely that this single-base change alone is a true variant that can serve as a meaningful differentiator separating ASVs.

### E. coli ASV fingerprints enable ID and tracking of strains across samples.

**FIG 5:**
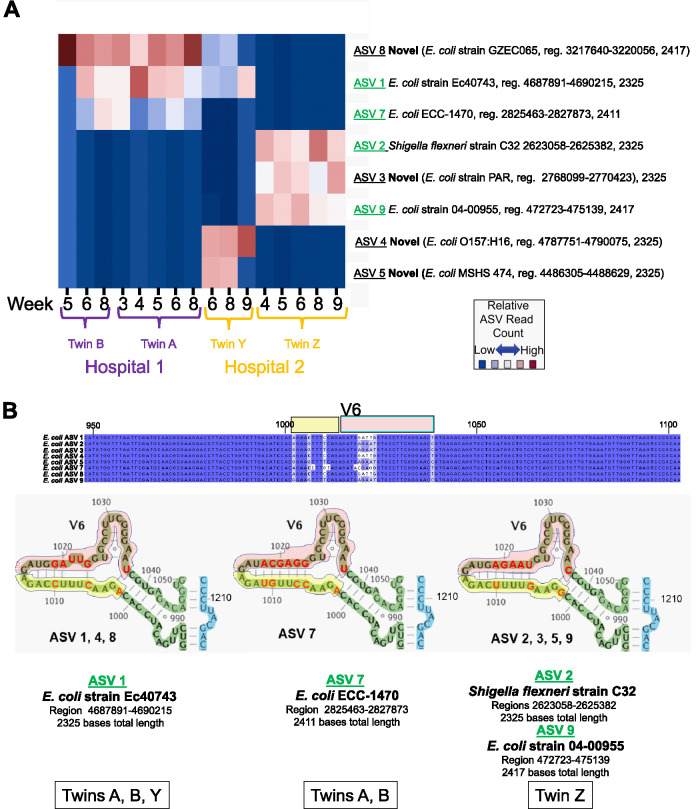
E. coli amplicon sequence variant fingerprints. (A) E. coli ASVs were inferred from longitudinal samples at the indicated weeks from reads classified as E. coli by SBanalyzer from twin pair A/B and twin pair Y/Z. Twins A and B were admitted simultaneously to hospital 1, and twins Y and Z were admitted simultaneously to hospital 2, as indicated. The 8 ASVs obtained from the samples using DADA2 were mapped to the NCBI database, and the closest-matching taxonomies were selected and are displayed to the right. ASVs in green text indicate perfect matches to the indicated NCBI region (reg.). “Novel” in bold text indicates an ASV that did not match perfectly to any sequence in the NCBI database but was present in multiple samples. The twin ID and the week that the sample was collected are indicated at the bottom. The relative number of each of the ASVs in each sample is indicated by shading, where dark red indicates more reads and blue indicates fewer. (B) E. coli ASV structure at the V6 stem-loop. E. coli ASVs were inferred from twin pair A/B and twin pair Y/Z using DADA2, and the eight resulting ∼2,300- to 2,400-base 16S-ITS-23S ASVs were aligned against each other in Jalview (40). A portion of the alignment from bases 948 to 1103 containing the V6 region is shown, with identical sequences across all ASVs in black text with dark blue highlighting and ASV-specific variations shaded in lighter colors. The yellow and red boxes above the alignment indicate the V6 stem-loop sequences shaded in the structures pictured immediately below the alignment in the middle. The 2-D structures of the aligned V6 regions of each ASV sequence were mapped to the E. coli 16S gene structure (41) from bases 988 to 1050; bases were numbered according to the alignment. Variant sequences from the alignment are depicted in bold red text. Yellow and red shading in the structures corresponds to the yellow and red boxes above the aligned sequences. The ASVs containing the exact stem-loop V6 sequence are listed below each structure. ASVs with NCBI BLAST searches that produced perfect matches to the entire V6 sequence are listed immediately below the structure in bold green underlined text. The NCBI BLAST strain ID, genomic region, and ASV length for the perfect full-length ASV match containing the V6 sequence are listed immediately below the bold green text. The individual twin samples containing ASVs that include each V6 sequence are listed at the bottom in the outlined boxes.

E. coli commonly occurs in human microbiome samples, and this species includes a variety of strains that can vary in impact from benign to pathogenic. DADA2 was used to infer ASVs from reads that SBanalyzer identified as E. coli from the four twin samples A and B and Y and Z, and the resulting fingerprints were compared across all time points in Fig. 5A. The E. coli ASV fingerprint results reveal findings similar to those for *Klebsiella* and *Enterobacter*, where the one ASV fingerprint was present in twins A and B and was distinct from the ASV fingerprints identified in twins Y and Z. Twin A was colonized at week 3, whereas twin B was colonized 2 weeks later. However, twins Y and Z had two completely different E. coli ASV fingerprints. Twin Y samples contained unique ASV 4 and ASV 5, whereas twin Z samples contained ASVs 2, 3, and 9. The fact that no ASVs were shared by twin Y and twin Z is evidence that they were colonized by different E. coli strains, even though they were admitted to the same NICU at the same time.

The sequences of the 8 E. coli ASVs obtained from the 16 longitudinal samples from 4 twins were compared to the E. coli 16S rRNA 2-D structure; the sequences in the V6 region are shown in Fig. 5B. The fact that each of the novel ASVs was identified at multiple time points, and ASV 7, ASV 8, and ASV 1 were identified in multiple individuals as subsets of a unique group of ASVs, demonstrates that the novel sequences are unlikely to be artifacts. The sum of these observations supports the hypothesis that novel ASVs can be a combination of sequences/structures shared with other bacterial 16S sequences and that DADA2 is capable of inferring very large 16S-ITS-23S ASVs with single-base resolution.

**FIG 6:**
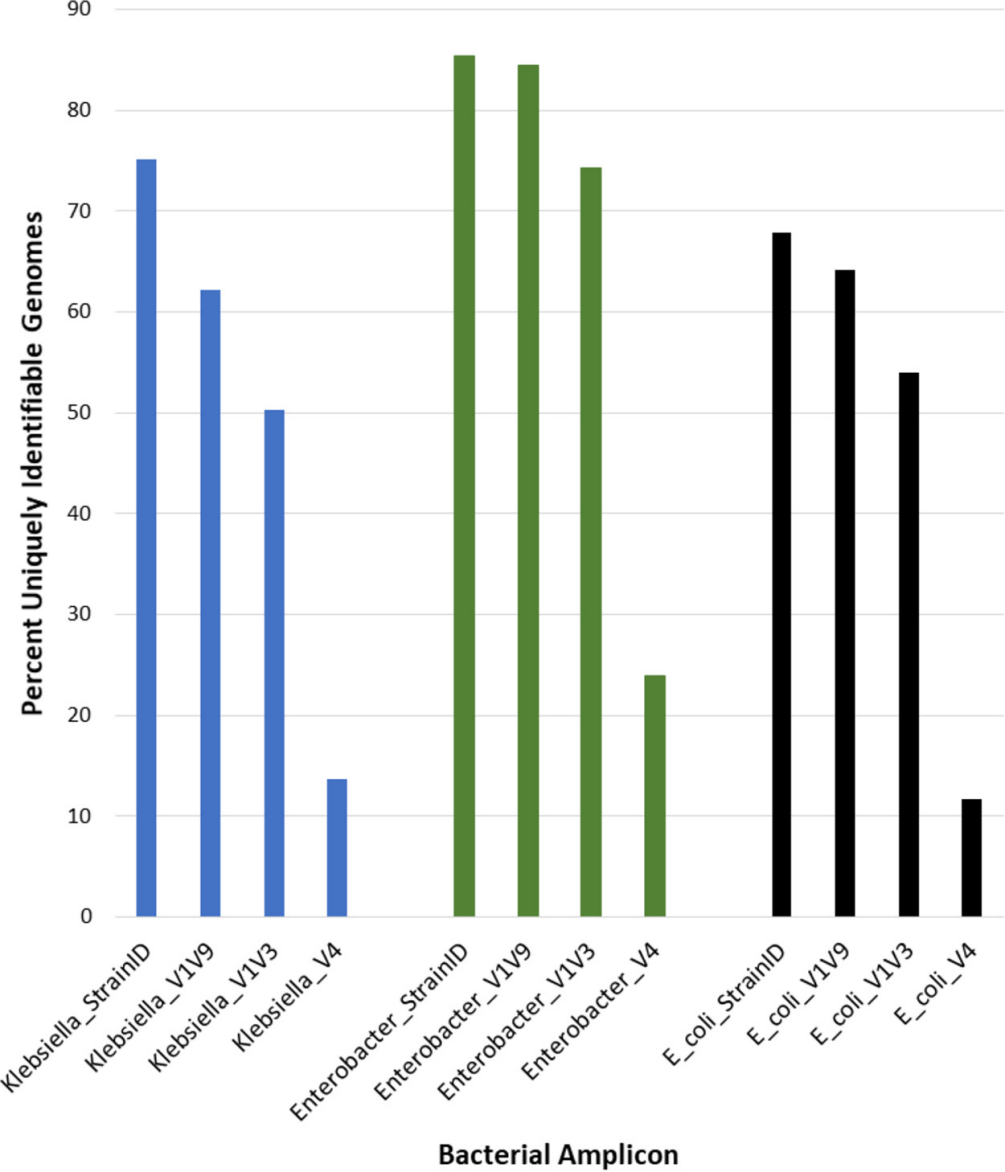
Uniquely identifiable *Klebsiella*, *Enterobacter*, and E. coli genomes in the Athena database. Amplicon sequences were compared for 458 *Klebsiella*, 109 *Enterobacter*, and 187 E. coli genome entries in the Athena database. Each *Klebsiella* and *Enterobacter* genome ID contained up to eight contiguous 16S-23S gene pairs, and E. coli contained up to seven, from which the sequences of the V4, V1-V3, V1-V9, and StrainID amplicons were extracted. A genome ID was considered unique if it contained either a unique amplicon or a unique combination of amplicons. The percentage of uniquely identifiable genomes is shown on the *y* axis, and the taxonomy and amplicon type are shown on the *y* axis.

Interestingly, twin Y shared E. coli ASV 1 and ASV 8 with twins A and B. A search of the Athena database showed that there are a number of 16S-23S regions shared across E. coli strains, but there are few sequenced strains that share all regions (Fig. 6). The fact that twins A and B and twin Y share only a subset of ASVs, and they were admitted to different hospitals at different times, indicates that they acquired different E. coli strains that share a subset of ASVs. It remains possible that these strains were present at very low levels and that a greater sequencing depth was required to detect these ASVs. The illustration *in silico* and in infant fecal samples is an indication that the variation in the combination of the ASVs (ASV fingerprint) provides sufficient resolution to enable strain-level tracking of bacterial transmission for closely related bacteria in hospital environments.

### *Klebsiella* reference database genome taxonomy investigation.

Next, we wanted to assess the accuracy of the species identification using the Athena database. Identification of bacterial taxonomies using either sequencing read data or ASVs requires that data be accurately mapped to a database containing correct taxonomies. The Athena database was created from well-sequenced genomes in the public domain, but it has been documented that there are many misidentified genomes in GenBank, even though best practices have been suggested for GenBank submissions (21). Additionally, existing entries in a database may not be updated as nomenclature is refined over time, so closely related species that were described at different times can sometimes be assigned different taxonomies based on the best practices upon publication (22). To address the potential for “misassignment” of taxonomies for sequenced genomes, three methods were used to explore the “correctness” of *Klebsiella* taxonomies within the Athena database, two based on 16S and one based on whole-genome sequences. The Athena database includes 458 *Klebsiella* genomes with 3,635 regions. In Fig. S2A and Fig. S2B, a randomly selected total of 1,387 *Klebsiella* full-length 16S genes and 16S-23S StrainID amplicons were extracted from the Athena database, and phylogenetic trees were generated to assess how well the taxonomies matched the tree topology. The phylogenetic tree for the 16S rRNA genes indicated that the different *Klebsiella* spp. mostly clustered as expected, with K. oxytoca and *K. michiganensis* being somewhat interleaved but mostly separated from Klebsiella aerogenes and K. pneumoniae. In contrast, in the StrainID phylogeny, several species grouped in distant clades, suggesting that the similarities in the tRNA composition of the ITS region had a greater effect on the tree topology than the 16S or partial 23S rRNA; however, this tree also had a greater distance between individual leaves. This suggests that the longer StrainID amplicon enables improved differentiation of amplicons from similar organisms.

**FIG 7:**
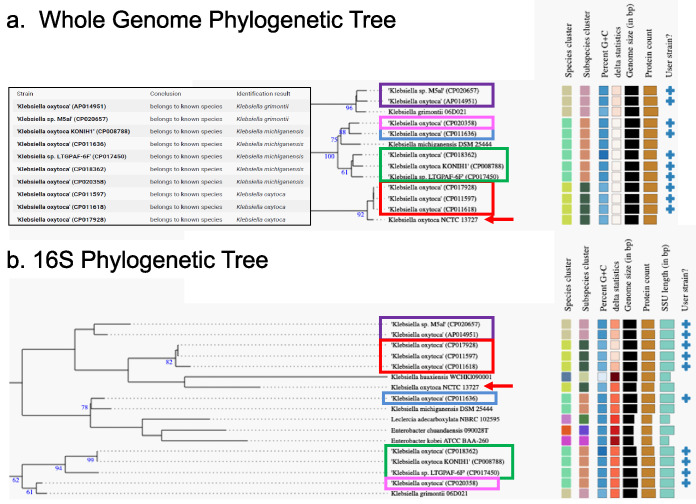
Whole-genome and 16S phylogenetic analyses. Ten K. oxytoca strains represented in the Athena database were compared with 11,300 strains in the TYGS database to identify closely related strains. The partial trees shown include all branches needed to place the 10 “user strains” selected from the Athena database. Colored boxes are used to indicate groups of genomes that sorted together. (a) Whole-genome phylogeny. The inset box indicates how the input Athena strains were identified by TYGS. The tree with detailed relationships was inferred with FastME 2.1.6.1 (37) from GBDP distances calculated from genome sequences. The numbers above branches are GBDP pseudobootstrap support values of >60% from 100 replications, with an average branch support of 85.5%. (b) 16S phylogeny tree inferred with FastME 2.1.6.1 from GBDP distances calculated from 16S rRNA gene sequences. The numbers above branches are GBDP pseudobootstrap support values of >60% from 100 replications, with an average branch support of 53.3%. GenBank accession numbers are in parentheses. SSU, small subunit.

To assess the taxonomic designation accuracy of the Klebsiella oxytoca sequences in the Athena database, 10 genomes labeled Klebsiella oxytoca with 16S-23S regions represented in the Athena database were compared to the TYGS database using both the 16S sequences and the full genomes (Fig. 7). The whole-genome phylogeny revealed that two of the “Klebsiella oxytoca” strains from the Athena database were closest to *K. grimontii*, five were closest to *K. michiganensis*, and only three were most closely related to K. oxytoca. The relationships inferred by the 16S rRNA gene phylogenetic tree were similar, with three strains being most similar to K. oxytoca NCTC 13727, but four of the five strains most closely related to *K. michiganensis* grouped differently. The two *K. grimontii*-related strains from Athena were sorted together as with the whole-genome results but far from the *K. grimontii* strain from the TYGS database. The differences between the whole-genome and 16S methods indicate that the 16S phylogeny may not always reflect the whole-genome phylogeny and that the taxonomic names in the databases are not always correct.

**FIG 8:**
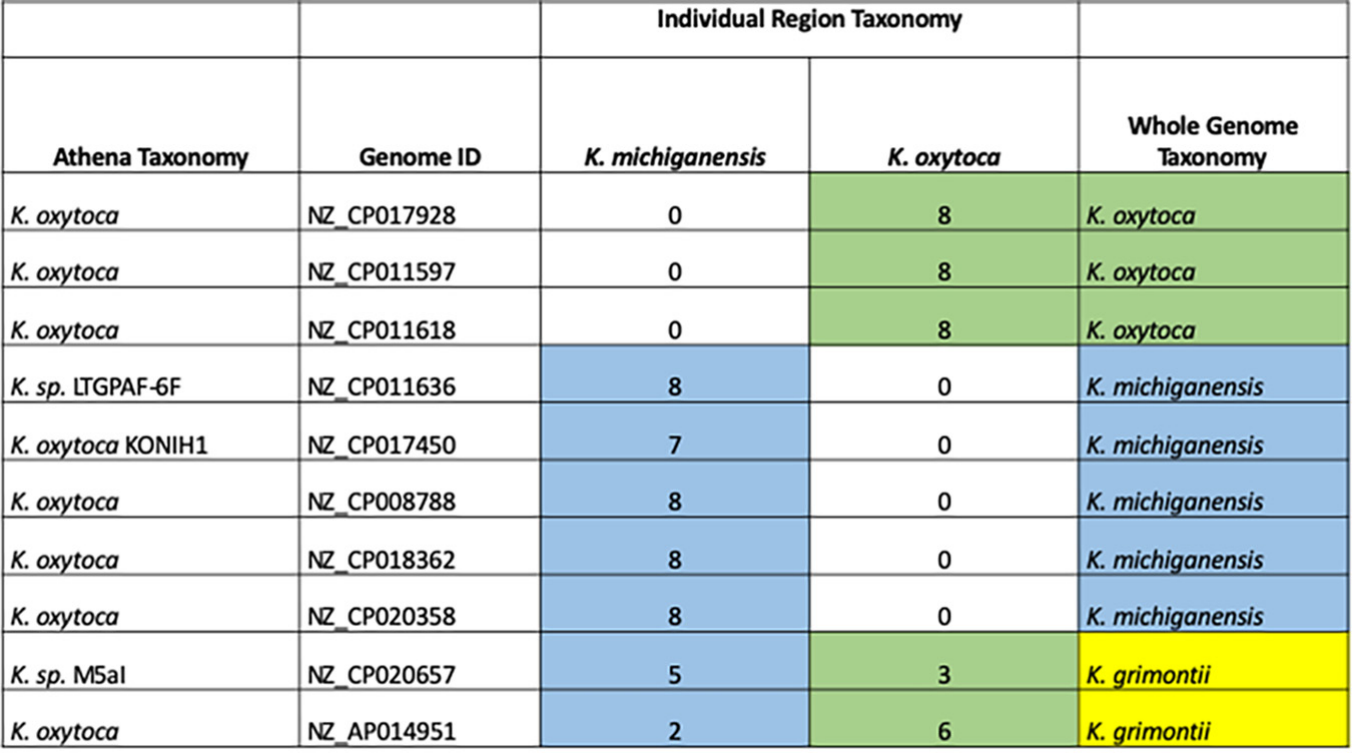
Individual 16S region taxonomies of 10 *Klebsiella* genomes from the Athena database. The first and second columns show the Athena taxonomic assignment and the unique genome ID (GenBank accession number). Each of the 7 to 8 16S regions from each genome ID was mapped individually. A total of 670 sequences were aligned using Clustal Omega (30) and manually curated before the phylogenic relationship was reconstructed using RAxML (31). The phylogenetic tree was annotated in iTOL (32), resulting in an individual region taxonomy for each that was either *K. michiganensis* or K. oxytoca, which were totaled in the appropriate columns. The whole-genome phylogeny taxonomy obtained from TYGS (33) is shown in the last column.

We further explored the discrepancy of the 16S rRNA and whole-genome phylogenies by examining the individual regions from each of the 10 *Klebsiella* strains from the phylogenetic analysis. All of the 16S rRNA gene copies were mapped individually to determine the taxonomy assigned to each 16S rRNA gene (Fig. 8). As expected, the genomes labeled as either *K. michiganensis* or K. oxytoca by whole-genome TYGS phylogeny contained regions that all mapped to the corresponding organism. Interestingly, *K. grimontii* genomes contain a blend of regions common to both *K. michiganensis* and K. oxytoca. This result indicates that although the taxonomy of a genome may not be up to date or the 16S rRNA gene phylogeny may not reflect the taxonomy, an understanding of the 16S gene combinations for organisms of interest provides an additional method for the discrimination of closely related taxonomies.

### Amplicon length correlates with utility for taxonomic discrimination.

The widely used ∼250-base V4 region of the 16S rRNA gene usually allows the identification of the bacteria to the family or genus level. A V1 through V9 (V1-V9) ∼1,500-base 16S rRNA gene amplicon can frequently yield taxonomic specificity at the genus or species level (6), but closely related bacterial strains may have identical 16S genes. In order to compare the abilities of different amplicons to uniquely identify a set of known genomes, an *in silico* comparison of 458 *Klebsiella*, 187 E. coli, and 109 *Enterobacter* strains was performed for amplicons covering the V4, V1-V3, V1-V9, and 16S-23S StrainID regions (Fig. 6). The trends for all three bacterial taxonomies demonstrate that for a given genus and species, most V4 amplicons are identical, whereas most StrainID amplicons are unique. Figure 6 shows that only 14% of the *Klebsiella* genomes could be uniquely identified using the ∼300-base V4 region amplicon. The ∼526-base V1-V3 amplicon sequences enabled the unique identification of approximately one-half of the genomes in the Athena database. The full ∼1,500-base 16S gene V1-V9 amplicon enabled the unique identification of 62% of the genomes, whereas the StrainID amplicons enabled the unique differentiation of 75%. This number of uniquely identifiable genomes may be an underestimate because the Athena database may contain duplicate genomes originating from the same strain that were submitted under different names or accession numbers.

## DISCUSSION

One of the major challenges of microbiome studies is the limited taxonomic resolution that the short stretches obtained by traditional 16S rRNA amplicon sequencing provide. Amplification and sequencing of the ∼2,500-bp 16S-23S StrainID portion of the rRNA operon and comparison of the sequences to the genome-based Athena database allow the identification of bacterial species and known strains. DADA2 can be used to reveal strains with unique amplicons or unique amplicon combinations that generate correspondingly unique ASV fingerprints. We tested this novel approach in premature infants who harbor a simple microbiome by analyzing longitudinal time series of fecal microbiome profiles from individual infants and pairs of twins.

Multiple strains of *Klebsiella* spp., E. coli, and *Enterobacter* spp. were detected in fecal samples of the five infants in this study. The variable ASV sequences inferred using DADA2 could be assigned to the 2-D structure of the 16S molecule, demonstrating that the base changes were not random and that they tended to vary such that the 2-D hairpin loop structures of known variable regions were maintained. The ASVs created from the StrainID sequencing data provided species- and strain-level resolution of *Klebsiella* spp., E. coli, and *Enterobacter* spp.

The increased resolution provided by the combination of the StrainID amplicon, the Athena database, and the DADA2 ASVs was strongly supported by the experimental design, which included longitudinal time series of samples obtained over consecutive weeks, enabling independent confirmation of novel ASVs over time in independent sampling events. For example, the unique ASV fingerprints from the stool samples from twin pair A/B indicate that they shared one Enterobacter cloacae strain, one Klebsiella pneumoniae strain, and one E. coli strain across multiple time points, while twin pair Y/Z carried the same *K. grimontii* strain, two distinct E. coli strains, and no *Enterobacter* strains. The E. coli results also demonstrate that the total complement of StrainID amplicons or the ASV fingerprint is sometimes necessary for differentiating closely related bacteria and revealing colonization patterns and temporal microbiome dynamics.

Although the StrainID amplicon generally enables higher-resolution taxonomic classification than the 16S gene alone, there are a number of limitations that should be considered. For example, even if ASVs obtained from a sample match a known strain, this does not necessarily confirm that the genome in the database is an identical match because closely related bacteria may share an identical set of StrainID amplicon fingerprints while harboring important differences elsewhere in the genome. While unique ASV fingerprints indicate real genomic differences, those differences may be limited to a few bases or even a single base in an amplicon and should be considered in the context of, for example, whether the differences are consistent across multiple individuals or time points. Ideally, experiments should be designed with technical repeats or longitudinal sampling such that conclusions are not based on variants identified in a single sample. Yet another important consideration is that not all ASVs will necessarily be represented at ratios corresponding to their relative genomic copy number because the distance of ribosomal operons relative to the origin of replication can drive increases or decreases of relative copy numbers as during replication. Genes close to the origin of replication will be present at a higher copy number than those closer to the terminator, especially if the cells were growing rapidly at the time when the sample was acquired (23). As a result, ASVs from known strains may be missed if the sequences are underrepresented due to growth and environmental effects. Genomic structure is another important consideration for the StrainID amplicon; there are species important in human gastric samples (e.g., *Helicobacter*) or environmental samples (e.g., certain *Planctomycetes*) where the 16S and 23S genes are thousands of bases apart, so no amplicon will be generated from these bacteria. In cases where genomes without proximal 16S-23S genes are prevalent, a lower-resolution 16S amplicon such as the V1-V9 region may be preferred.

Our investigation into *Klebsiella* taxonomic assignments using publicly available genomes reveals that it is important to consider whether the taxonomies that are attached to the best sequence matches in a database were correctly assigned and whether any important updates to the classifications were made after the sequences were posted. Care should be taken in the assignment of species- and strain-level classification of newly discovered bacteria so that new assignments are as correct as possible.

In summary, the StrainID rRNA amplicon provides a higher level of taxonomic information than the full 16S gene and can therefore provide a higher-resolution taxonomic picture of the overall microbiome. *Klebsiella*, *Enterobacter*, and E. coli were used as examples to demonstrate the ability to differentiate closely related taxonomies of specific early microbiome colonizers in infants confined to the NICU. Similar methods can be used to obtain high-resolution ASVs for other target bacteria using the StrainID amplicon. The assay provided a high-resolution longitudinal view of the colonization of the gut in premature infants, which may be useful for tracking the results of therapeutic interventions as well as opening a window into understanding how commensals are established in the human microbiome after birth. It may be possible to use the assay to identify improvements to treatments that establish important commensals earlier for improved health outcomes over short-term hospital stays and long-term development. The StrainID amplicon provides a rapid, practical, high-throughput, and high-resolution method of identifying and tracking known and unknown pathogens and commensal bacteria in the complex environment of the gut microbiome.

## MATERIALS AND METHODS

### Experimental design.

Longitudinal fecal samples from seven infants, including two sets of premature twins, born at gestational ages close to 30 weeks and cared for in two different NICUs, were collected each week from admission to the NICU to discharge. Metadata for each infant were collected concurrently, including antibiotic treatment, any diagnosed infections or dysbiosis, and nutritional sources. The bacterial content of infant fecal material was analyzed using the ∼2,500-base StrainID amplicon to determine whether strain-specific amplicon sequences could be used to monitor the development of microbiomes in the infants at high resolution. The design also included *Klebsiella* isolates obtained from infants previously treated in the NICU as strain-level controls for the analysis, to determine whether the StrainID amplicon could be used to differentiate the isolates and whether signals from the same isolates could be identified in the study.

### Fecal sample isolation and PCR.

Preterm infant fecal samples were obtained from subjects cared for at two affiliated NICUs in Hartford and Farmington, CT. Infants were enrolled during 2018 as part of an ongoing neonatal microbiome study approved by the Institutional Review Board at Connecticut Children’s Medical Center. Infant fecal samples were collected on an approximately weekly basis beginning with the first bowel movement until discharge, using sterile disposable spatulas during diaper changes; placed into sterile containers; and immediately frozen at −80°C until processing. Approximately 1 to 5 mg of fecal material was used as the input into the StrainID kit (StrainID set A [barcodes 1 to 96]; Shoreline Biome). Fecal sample DNA was isolated according to the manufacturer’s instructions. Briefly, 50 μl of reconstituted lysis reagent was added to each sample. Subsequently, 50 μl of a 0.4 M KOH solution was added, and the samples were heated to 95°C for 5 min to lyse the cells. The plate was spun briefly to pellet the fecal debris, and 50 μl of the supernatant was transferred to a clean plate. Fifty microliters of DNA purification beads was added, the DNA was allowed to bind, and the pellets were washed with 70% ethanol. DNA was eluted in Tris-EDTA (TE), and 10 μl of eluted DNA was transferred to the corresponding well in the PCR plate. PCR mix (2×) was added, and PCR was performed according to the manufacturer’s instructions to amplify and barcode each sample. Samples were pooled and purified via MinElute spin columns (catalog number 28004; Qiagen).

### Individual Klebsiella oxytoca isolates.

Klebsiella oxytoca fecal isolates were obtained from nonrelated preterm infants who developed necrotizing enterocolitis (14). Individual colonies were screened by colony PCR for *pehX*, a genetic marker specific for K. oxytoca (24). For whole-genome sequencing, genomic DNA was extracted using the MasterPure DNA purification kit (Lucigen) and used for Nextera XT (Illumina)-based amplicon library preparation and sequencing on the Illumina platform at the Microbial Analysis, Resources, and Services (MARS) facility at the University of Connecticut.

### StrainID amplicon and 16S gene sequences.

The StrainID amplicon spans the full 16S and partial 23S rRNA genes. Forward and reverse primers were synthesized as a 3-part sequence, as follows: 5′–adaptor–barcode–target-specific primer–3′. The 16S adaptor sequence is 5′-GGTTATGCGGTTCACTGC-3′. All barcodes were selected from the list of 384 Pacific Biosciences (PacBio)-recommended barcodes (https://www.pacb.com/products-and-services/analytical-software/multiplexing/). The target-specific forward primer sequences used are a pool of primers with the sequence 5′-AGRRTTYGATYHTDGYTYAG-3′. The reverse primer had a similar 5′–adaptor–barcode–target-specific primer–3′ structure, where the adaptor sequence is 5′-CGTCACTTGGCGTATTGG-3′, and the target-specific sequences are a pool with the sequence 5′-AGTACYRHRARGGAANGR-3′. The forward primer is located at the start of the 16S gene, whereas the 23S primer site is located about 600 bases inside the gene. Both primer sites were used as a starting point to identify additional primer site variants that exist in bacteria found in the following databases: Shoreline Biome Athena, SILVA 16S and 23S (25), and Riken (43).

For the 16S rRNA gene sequences extracted from the databases for *in silico* comparisons, the reverse primer used was based on the 1492r primer: 5′-TASVGHTACCTTGTTACCGACTT-3′.

### DNA sequencing.

Amplicon libraries were created using the SMRTbell express template prep kit 2.0 (catalog number 100-938-900; PacBio) according to the manufacturer’s instructions. The library was sequenced on a Sequel 1 system (Pacific Biosciences) at the University of Delaware, Delaware Biotechnology Institute Sequencing and Genotyping Center, Newark, DE. A total of 320,054 circular consensus (ccs) reads were produced using default settings.

### Taxonomic assignment of reads.

SBanalyzer 2.4 (Shoreline Biome) was used to map ccs reads to the Athena database and assign taxonomic identification to all reads. SBanalyzer produces a summary comma-separated values (“.csv”) file with bacterial taxonomic ID and the corresponding read count for all samples that can be manipulated in Excel, LibreOffice, or other spreadsheet programs as well as a “.taxonomy” file (see Data File S3 at https://github.com/joerggraflab/ShorelineBiomeStrainID-files) with the taxonomic assignment and a “.groups” file (see Data File S4 at the URL mentioned above) with the sample assignment for each read. SBanalyzer is a graphical user interface-based pipeline that encapsulates custom algorithms and external calls to version 1.40.5 of mothur (26). However, the ∼2,500-bp amplicon reads required significant changes to the default mothur methods for both demultiplexing and mapping for optimal results. Demultiplexing of barcodes and assignment of reads to samples employed custom code optimized for 16-base PacBio barcodes combined with the dual-unique barcode structure of the primers, which was able to assign 97 to 99% of ccs reads to a sample. In addition, mothur was customized to use BLAST-plus (27) to map the ∼2,500-bp amplicon reads since the standard mothur algorithms and settings failed to correctly assign long amplicon taxonomies.

### Athena database.

The Athena database is an integrated part of the SBanalyzer pipeline that contains contiguous 16S-23S sequences. The Athena database was created from bacterial genomic data downloaded from RefSeq on 21 May 2019. A total of 5,551 “reference” and “representative” genomes along with 13,634 other genomes assembled at the “complete” and “chromosome” levels were downloaded, for a total of 19,185 genomes. The NCBI taxonomy database was used to annotate the RefSeq sequences. Target regions were extracted using a merger of NCBI’s GFF genome annotations and *de novo* annotations from Barrnap v0.8. Regions were defined by pairs of neighboring 16S and 23S genes that are between 2,000 and 8,500 bp. If a match for a read to a 16S-23S region in Athena, with errors included, is >97% and has a higher score than other matches, the read will be identified as the matching strain in the database. If a read matches equally well (or poorly) to multiple regions in the database, or if there is no match at the strain level, taxonomy will be reported at the highest level possible where an unambiguous call can be made. A similar cutoff is made at the species level, with a threshold of 95%. As a result, a novel *Klebsiella* read with 96% identity to an existing strain would be reported as “*Klebsiella_oxytoca*_unclassified,” and a novel *Klebsiella* species would be reported as “*Klebsiella*_unclassified.”

### Athena database phylogenetic categorization.

A program was created to load the NCBI taxonomy flat files into a searchable data structure. The data have a hierarchical structure, so a genome’s GFF3 file would typically provide a strain-level taxonomic classification for the genome, and the node representing that strain in the taxonomy database would have a parent. The chain hierarchy can be followed to the tree root to determine the full taxonomic classification. Most nodes in the taxonomy database have multiple names, so the “best” name needed to be selected using heuristics. Also, some tree nodes have nonstandard phylogenetic-level categorizations such as “superfamily” or “subgenus,” so some harmonization was performed to map chains of nodes to the typical levels as much as possible: kingdom, phylum, class, order, family, genus, species, and subspecies. This enabled the database output to assign taxonomic classification to specific levels such as “level 6” while providing phylogenic consistency across samples. The program also modified the taxonomy data to be more compatible with mothur (26). The program was used to create the .taxonomy file associated with the database that maps each sequence identifier to a multilevel phylogenetic classification.

### Mapping to the Athena or SILVA database.

Database selection is integrated into the automated SBanalyzer pipeline. There are two choices in the drop-down menu for mapping reads, the Athena database and the SILVA database (25).

### Decontamination of *Delftia* species reads.

Almost all samples analyzed for StrainID amplicon sequences, including negative controls, appeared to have some level of Delftia tsuruhatensis contamination. *Delftia* is a known contaminant of laboratory water supplies (28, 29). *Delftia* could have been introduced in water used to dissolve the KOH lysis solution or in the TE used to elute DNA during sample preparation. The specificity of the StrainID amplicon enables the identification and removal of contaminating *Delftia* reads. *Delftia* reads called by SBanalyzer were confirmed by comparing *Delftia* sequences in the samples to all five 2,639-bp 16S-23S rRNA genes from Delftia tsuruhatensis strain CM13 (GenBank accession number NZ_CP017420.1) present in the Athena database to determine how closely reads mapped. Reads were approximately 99.8% identical to the published genome and similar across all contaminated samples, indicating that the *Delftia* contaminant was highly related to the sequenced strain. These *Delftia* reads were removed from the analysis.

### DADA2 inference of amplicon sequence variants.

DADA2 is described for use with PacBio reads (10) and was installed according to the instructions at https://benjjneb.github.io/dada2/dada-installation.html. FASTQ files were demultiplexed for each sample using SBanalyzer, with the “NoTrim” option in the SBanalyzer drop-down menu. Reads assigned “*Klebsiella*” taxonomy by SBanalyzer in the .taxonomy file output were used as the input for DADA2. The “R” script in Data File S1 at https://github.com/joerggraflab/ShorelineBiomeStrainID-files was used to perform read processing and specify the DADA2 parameters for ASV inference. The output includes two .png files containing a sequence table heat map and read length plots, a .FASTA file containing the amplicon sequence variant sequences, and two R objects containing the workspace file and the DADA2 output file. Demultiplexed FASTQ files were filtered on the basis of taxonomy assigned by SBanalyzer, selecting only reads at the desired taxonomic level (for example, “*Klebsiella*”), using a custom Python “readfinder” script (see Data File S2 at https://github.com/joerggraflab/ShorelineBiomeStrainID-files).

The selected reads from each sample were primer trimmed and filtered to reads within the length range of 1,900 to 3,000 bp. The sequences within this length range were then dereplicated and passed to DADA2 to build an error model for read correction. The trimmed and filtered reads were analyzed manually via a histogram of the read lengths of all samples to identify peaks of read lengths that are likely to represent unique amplicons. The corresponding read length ranges (i.e., the 2,400- to 2,405-bp range from each sample) were passed to DADA2 and pooled for ASV inference. A sequence table of ASV abundance per sample was produced as part of the DADA2 output, and a heat map was generated in R using the sequence table. Taxonomic information was added manually to the sequence table used to build the heat map.

ASV sequences for E. coli, *Klebsiella*, and *Enterobacter* are listed in Data Files S5 to S7, respectively, at https://github.com/joerggraflab/ShorelineBiomeStrainID-files.

### rRNA phylogenetic analysis.

*Klebsiella* StrainID sequences were exported from the Athena database and imported into Geneious Prime version 2020.1 (Biomatters). Within Geneious, the sequences were aligned using Clustal Omega (30) and manually curated. The phylogeny was inferred using RAxML (31). The tree was annotated in iTOL (32).

### Determination of closely related type strains.

Determination of the genomes of the closest type strains was done in two complementary ways. First, all user genomes were compared against all type strain genomes available in the TYGS database (33) via the MASH algorithm, a fast approximation of intergenomic relatedness (34), and the 10 type strains with the smallest MASH distances were chosen per user genome. Second, an additional set of 10 closely related type strains was determined via the 16S rRNA gene sequences. These were extracted from the user genomes using RNAmmer (35), and each sequence was subsequently subjected to a BLAST search (27) against the 16S rRNA gene sequence of each of the currently 11,300 type strains available in the TYGS database. This was used as a proxy to find the 50 best-matching type strains (according to the bit score) for each user genome and to subsequently calculate precise distances using the genome BLAST distance phylogeny approach (GBDP) under the algorithm “coverage” and distance formula d5 (36). These distances were finally used to determine the 10 closest type strain genomes for each of the user genomes.

### Pairwise comparison of genome sequences.

All pairwise comparisons among the set of genomes were conducted using GBDP and accurate intergenomic distances inferred under the algorithm “trimming” and distance formula d5. One hundred distance replicates each were calculated. Digital DNA-DNA hybridization (dDDH) values and confidence intervals were calculated using the recommended settings of GGDC 2.1 (36).

### Phylogenetic inference.

The resulting intergenomic distances were used to infer a balanced minimum evolution tree with branch support via FASTME 2.1.4, including subtree pruning and regrafting (SPR) postprocessing (37). Branch support was inferred from 100 pseudobootstrap replicates each. The trees were rooted at the midpoint and visualized with PhyD3 (38).

### Type-based species and subspecies clustering.

The type-based species were clustered using a 70% dDDH radius around each of the 32 type strains (33). Subspecies clustering was done using a 79% dDDH threshold (39).

### Data availability.

Annotated whole-genome assemblies for K. oxytoca isolates were submitted to the NCBI BioProject database under BioProject accession number PRJNA608440. StrainID amplicon data for this study are deposited under BioProject accession numbers PRJNA663638 and PRJNA663575. The SRA accession numbers for the reads are SRR12647577 to SRR12647615 and SRR12692773 to SRR12692775. Supplemental data files are available at https://github.com/joerggraflab/ShorelineBiomeStrainID-files.
